# A Complex Case of Idiopathic Purulent Pericarditis in an Immunocompetent Adult

**DOI:** 10.7759/cureus.46930

**Published:** 2023-10-12

**Authors:** Faisal Aloqab, Lulwa Alsadah, Shanei Shanei

**Affiliations:** 1 Internal Medicine, Bahrain Defence Force Hospital, Riffa, BHR; 2 Pathology, Arabian Gulf University, Manama, BHR; 3 Cardiology, Bahrain Defence Force Hospital, Riffa, BHR

**Keywords:** pericardiocentesis, pericardial effusion, bacterial pericarditis, purulent pericarditis, acute pericarditis

## Abstract

Acute purulent pericarditis is a fatal complication of bacterial pericarditis. Purulent pericarditis usually arises secondary to an infection that spreads directly or hematogenously. The mortality rate reaches 100% in untreated purulent pericarditis. We present a case of complex idiopathic purulent pericarditis caused by Methicillin-sensitive *Staphylococcus aureus *(MSSA). In this report a 39-year-old male presented with shortness of breath, cough and chest pain. He was diagnosed with pericardial effusion with signs of cardiac tamponade. He underwent pericardiocentesis and aspirated pericardial fluid grew *Staphylococcus aureus. *He was started on IV antibiotics. However, he had a recollection of pericardial fluid and thus a pericardial window and removal of fibrinous material was done. He was treated with IV antibiotics for a total of seven weeks. High clinical suspicion is needed in diagnosing purulent pericarditis and prompt treatment helps in achieving favorable outcomes for the patient as demonstrated in our case.

## Introduction

Acute pericarditis is defined as an acute inflammation of the pericardium. In the modern antibiotics era, only 1-2% of acute pericarditis is bacterial in origin. The most common cause of acute pericarditis remains to be idiopathic, consisting of around 80-90% of the reported cases [1].

Acute purulent pericarditis is characterized by the presence of purulent fluid in the pericardial cavity. This complex and fatal complication of acute bacterial pericarditis requires high clinical suspension and prompt treatment. If it is left untreated the mortality approaches 100% [2].

Purulent pericarditis usually occurs secondary to an infection. The spread to the pericardium occurs either through hematogenous seeding or direct spread from the thoracic region. Organisms that have been implicated in purulent pericarditis include *Staphylococcus aureus*, *Streptococcus pneumoniae*, *Streptococci viridans*, *Haemophilus influenzae*, and anaerobic bacteria [3].

We hereby present a case of complex idiopathic purulent pericarditis caused by Methicillin-sensitive *Staphylococcus aureus* (MSSA), highlighting within it our approach to the management and review of the literature of alternative treatment strategies.

## Case presentation

A 39-year-old male, who is not a known case of any medical illness, presented to the emergency department (ED) with a two-week history of shortness of breath, dry cough, and pleuritic left-sided chest pain. This was associated with fatigability, night sweat, and weight loss over three weeks.

**Figure 1:**
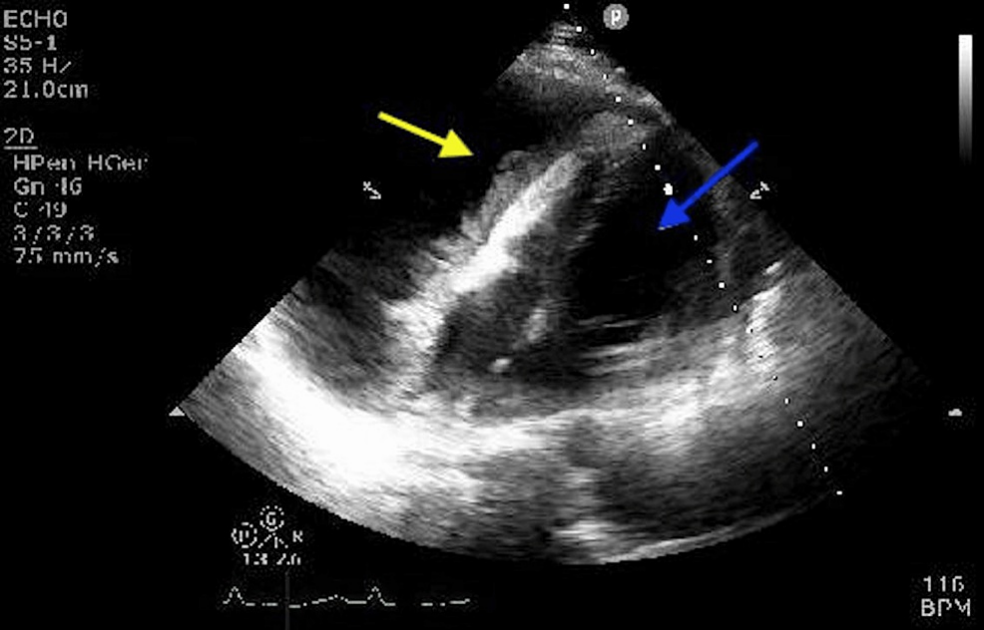
Echocardiogram image showing large pericardial effusion. Yellow arrow pointing to pericardial effusion with threads. Blue arrow pointing to left ventricle.

On arrival at the ED, he was hemodynamically stable and afebrile. A physical exam revealed bilateral lung basal crepitations and muffled heart sounds. His chest X-rays showed bilateral pleural effusion and cardiomegaly. An electrocardiogram (ECG) was done and showed a sinus rhythm with pulses alternant. Thus an urgent echocardiography was requested after the suspicion of pericardial effusion clinically and radiologically. Echocardiography showed a large pericardial effusion measuring 57 mm on the right side of the heart with signs of cardiac tamponade (Figure 1).

**Figure 2:**
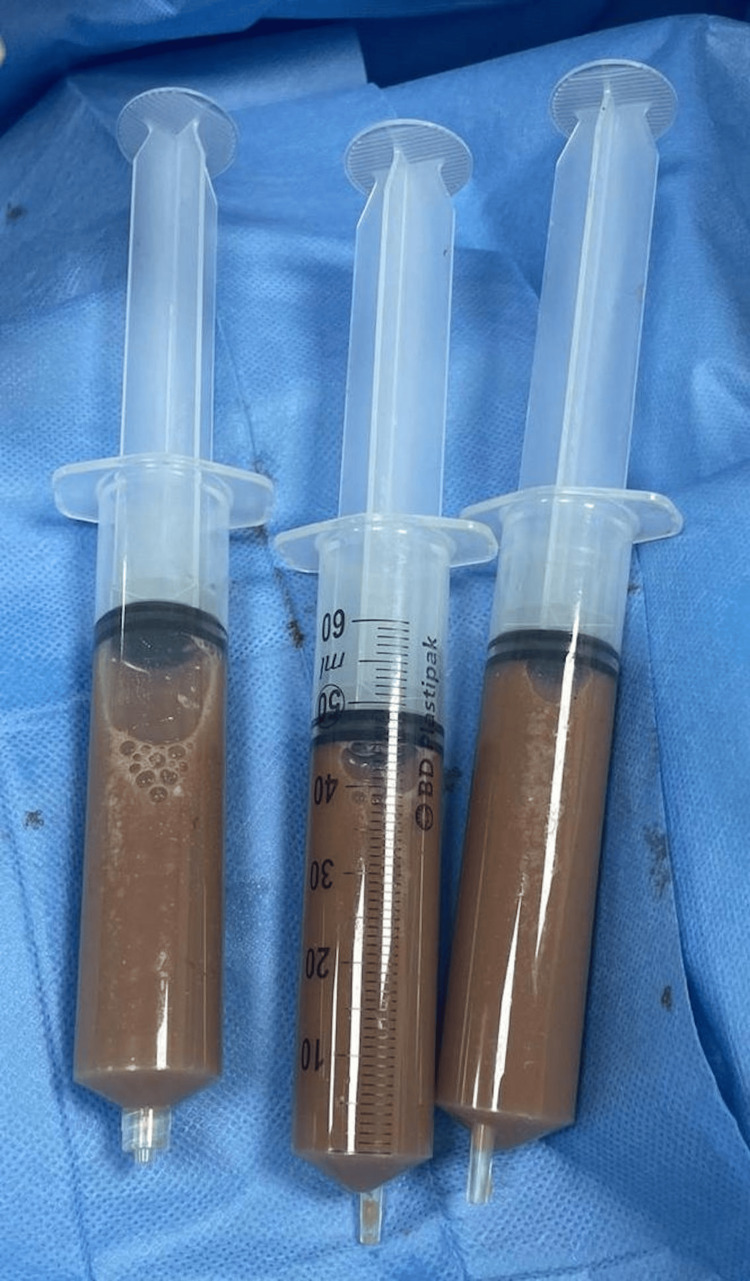
Syringes showing turbid purulent pericardial fluid following drainage

In view of the above findings, the patient underwent pericardiocentesis which showed immediate improvement in tamponade signs. A total of two liters of turbid, thick, purulent fluid was drained (Figure 2).

His labs showed a white blood cells (WBC) of 8.67 (neutrophils: 57%) and an elevated C-reactive protein (CRP) of 48.7. Analysis of pericardial fluid revealed 103,000 WBC (88% neutrophils) and 70,000 red blood cells (RBC). Pericardial-to-serum lactate dehydrogenase (LDH) ratio was 22.5 and the pericardial-to-serum protein ratio was 0.89. Pericardial fluid cytology showed acutely inflamed pericardial fluid with the absence of malignant cells. The culture of the aspirated fluid grew *S. aureus*.

Other investigations including blood, sputum cultures, and serology tests, including HIV were negative. The patient had no prior history of trauma to the chest wall, nor did he report a significant past surgical history. The patient underwent transesophageal echo (TOE) to rule out infective endocarditis. The TOE showed no visible vegetation or thrombus. After discussing with the infectious disease team with regards to the best choice of antibiotics we opted to start the patient on IV cefazolin.

**Figure 3:**
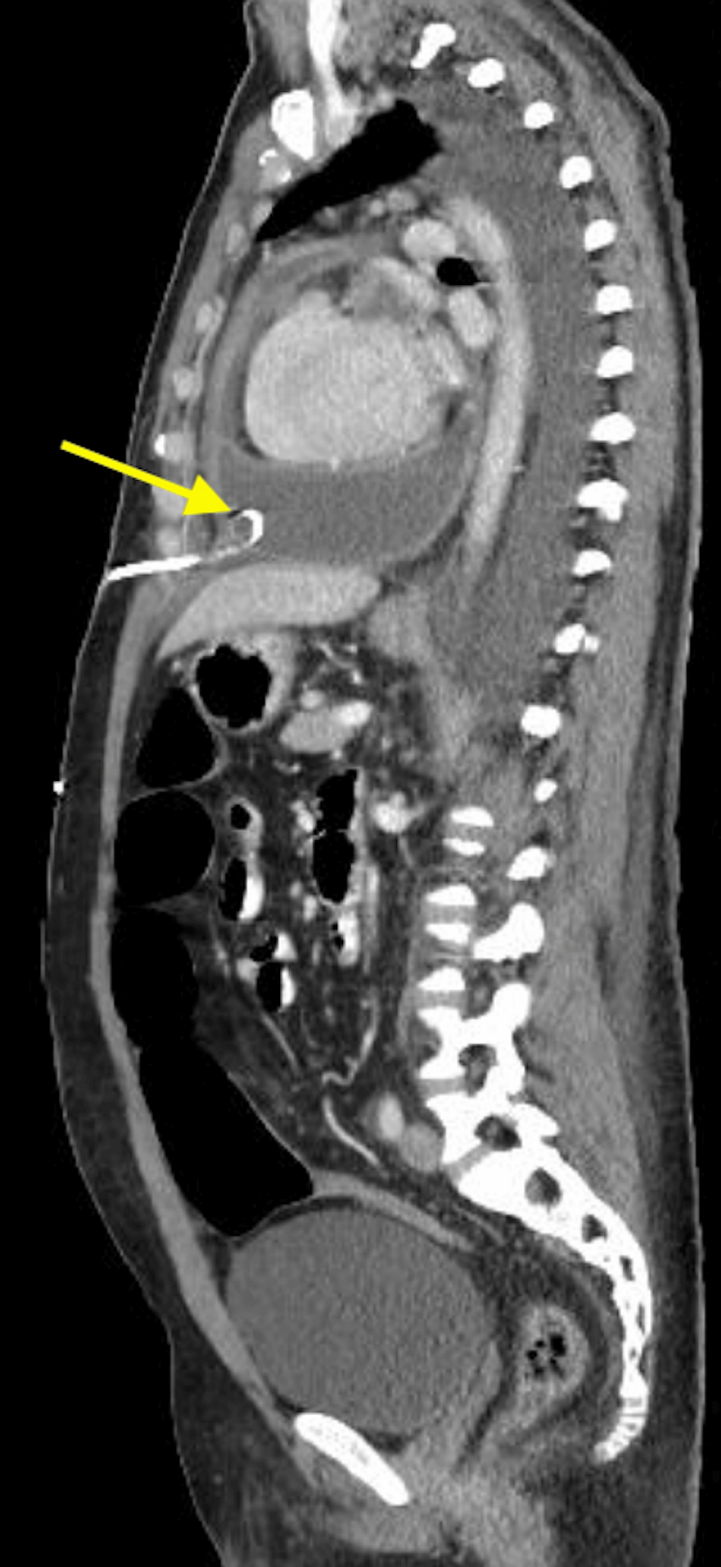
CT scan with contrast showing pericardial drainage catheter in situ in the pericardial space. Yellow arrow pointing to tip of the catheter in pericardial space surrounded by large pericardial effusion.

During his hospital stay, follow-up echocardiography and chest X-ray (CXR) showed incomplete resolution of pericardial fluid. Although he had subjective clinical improvement, we opted to do a CT scan of the chest. The CT of the chest showed cardiomegaly with mild to moderate pericardial fluid collection. A pericardial drainage catheter was seen in situ in the pericardial space, with no pericardial masses, a moderate left-sided pleural effusion, and a mild right-sided pleural effusion were also noted (Figure 3). After discussion with an interventional radiologist, drainage of the left-sided pleural effusion was done for both diagnostic and therapeutic purposes. The pleural fluid analysis showed a similar appearance and cytology of the pericardial fluid, however, the cultures remained negative.

In view of the recollection of pericardial fluid, the case was discussed in a multidisciplinary team meeting between the cardiology, cardiothoracic surgery, and infectious disease teams, and it was decided to take the patient for surgical intervention. Pericardial window and removal of fibrinous material were done. A single piece of fibrofatty yellowish tissue, measuring 30 x 20 x 18 mm and weighing 6g, was taken and sent for histopathological analysis. The biopsy shows a significant fibrinoinflammatory thickening associated with chronic active inflammatory cell infiltrate. No granuloma, atypia, or malignancy was seen.

Following the above, the patient showed signs of clinical, biochemical, and radiological improvement. He was treated with IV cefazolin for three weeks from the date of pericardial washout followed by four weeks of IV Rocephin. The patient was followed up in the clinic for six months and there were no signs of recollection of the pericardial fluid in the echocardiography and he has been doing well since.

## Discussion

Acute pericarditis is an inflammatory condition that involves the pericardium, with idiopathic and viral being the most common etiologies. Bacterial pericarditis represents a minority of cases, with *S. aureus* being one of the causative organisms. Bacterial pericarditis usually occurs from either a direct spread from intrathoracic infection or hematogenously.

In this report, we presented a case of idiopathic *S. aureus* pericarditis. While reviewing the literature we found few cases of patients presenting with idiopathic *S. aureus* pericarditis [4]. Mortality rates in such cases reach 100% if left untreated and 40% in treated patients [5].

Diagnosing bacterial purulent pericarditis poses a great challenge for many physicians who need to rely on the clinical presentation, investigations, and imaging available. Patients most commonly present with chest pain, however, a large retrospective study showed that patients can present with vague symptoms such as fever and chills. The physical examination can demonstrate findings such as pericardial rub and pulsus paradoxus. However, these were found in less than 50% of the patients presenting with pericardial effusion [1]. Laboratory findings such as elevated inflammatory markers can be seen, and more commonly with bacterial pericarditis an elevated neutrophils count can be observed in the cell differential. ECG will show diffuse ST-segment elevation, PR segment depression, electrical alternans, or low voltage criteria. An echocardiogram (ECHO) can show evidence of an increased amount of pericardial fluid in the pericardial space [2]. As the aforementioned investigations will not be able to differentiate it from other forms of pericarditis, given its aggressive nature, and high mortality rate, clinicians should have a high index of suspicion for purulent pericarditis, as aggressive treatment is required [3].

Immediate intervention is essential once purulent pericarditis is suspected. Drainage of pericardial space with placement of pericardial catheter is required, in addition to culture-specific antibiotics. In some cases, despite the immediate drainage, loculation with fibrin collection may result in incomplete drainage of the pericardial space. As a consequence of ineffective drainage, patients might develop constrictive pericarditis [6]. Therefore, other methods for evacuation are necessary to have complete drainage. These include fibrinolysis, subxiphoid drainage, wide-bore pericardiotomy, pericardial window, and pericardiectomy.

Administration of intrapericardial fibrinolytics is useful in cases with fibropurulant pericarditis. Streptokinase and streptodornase infusion into the pericardial space can stop the drainage without complications and prevent the development of pericardial constriction [7,8]. Once the drain is inserted, fibrinolysis should be done as fibrin accumulation increases during the first week of the disease. After two weeks, fibrosis starts formation when fibrinolysis has no effect [9].

Subxiphoid pericardiotomy is used for complete drainage by mechanical breakdown of septation. This method allows permanent drainage with the prevention of sternal and pleural contamination. Also, it is considered a less aggressive modality than thoracotomy. However, it has difficulties reaching posterior fluid loculations [10]. Another procedure that can be done is the pericardial window. It can be done either externally by sternotomy or pleuropericardial window, both of these procedures have a high risk of sternal infection and pleural contamination respectively [11].

A pericardiectomy is preserved for patients with refractory symptoms such as recurrent tamponade, persistent pericarditis, or constrictive pericarditis. Pericardiectomy has higher morbidity and mortality risk which can be explained by the fact that pericardiectomy is indicated in complex cases [12].

In our case, high clinical suspicion and good clinical judgment were present from the beginning which aided in the diagnosis of pericardial effusion. Following that appropriate investigations were done promptly. Consequently, the diagnosis was reached which guided the team to proceed with immediate drainage of the pericardial effusion, and starting the patient on appropriate antibiotics. The patient was then monitored closely allowing the team to detect the recollection early and the decision to go for a pericardial window was taken. This in turn allowed us to achieve an excellent outcome with our patient.

## Conclusions

Purulent pericarditis carries a high mortality rate. Early diagnosis and appropriate treatment help in improving the patient’s survival rate. We advocate for clinicians to remain vigilant in such cases and for their clinical suspicion to remain high. We propose early drainage of such effusions followed by IV antibiotics and close monitoring of the patient. In case of any recollection, we advise surgical intervention opting for a pericardial window as this was demonstrated in our case yielding a positive clinical outcome with our patient.
